# The Affective Nature of Formulaic Language: A Right-Hemisphere Subcortical Process

**DOI:** 10.3389/fneur.2018.00573

**Published:** 2018-07-24

**Authors:** Diana Van Lancker Sidtis, John J. Sidtis

**Affiliations:** Communicative Sciences and Disorders, New York University, New York, NY, United States

**Keywords:** formulaic language, (LH) damage, PET, RH damage, neurolinguistic

## Abstract

Formulaic expressions naturally convey affective content. The unique formal and functional characteristics of idioms, slang, expletives, proverbs, conversational speech formulas, and the many other conventional expressions in this repertory have been well-described: these include unitary form, conventionalized and non-literal meanings, and reliance on social context. Less highlighted, but potent, is the intrinsic presence of affective meaning. Expletives, for example, signal strong emotion. Idioms, too, inherently communicate emotional connotations, and conversational speech formulas allow for empathetic bonding and humor. The built-in affective content of formulaic expressions, in combination with their other unique characteristics, is compatible with the proposal that brain structures other than those representing grammatical language are in play in producing formulaic expressions. Evidence is presented for a dual process model of language, whereby a right hemisphere-subcortical system modulates formulaic language.

Connotations, affect, attitudes, and emotional meanings inhere essentially in formulaic language—fixed, unitary expressions that are known to a language community. Expletives (*Dammit, Good heavens*) make this point easily: their purpose is to communicate anger, surprise, shock, disapproval, or excitement (1–4). Idioms engage emotional arousal, subtle or strong, positive or negative. The idiom *he's out on a limb* communicates worry, risk, failure, and anxiety, while a matched literal sentence, *he's out in a boat*, is neutral. *Don't bite the hand that feeds you* carries a warning and a criticism; *He pulled the rug out from under us* implies disappointment, dismay, and reproachful anger. As a standard ingredient of their meaning—e.g., *Sleep with one eye open, In a nutshell, He's at the end of his rope, Just in the nick of time, Quit cold turkey, Shoot for the stars, You lucky dog, You're playing with fire, She has a snowball's chance in hell, I'll be there with bells on*—formulaic expressions weave together affect and attitude, which may be empathetic, reproachful, suspicious, or encouraging. Similarly, conversational speech formulas (*Okay!, Right!, Really? You're kidding!, Gotcha!; Whatever!; Go to hell; Knock on wood; It's all good; Shut your cakehole!*) carry connotations of affirmation or rejection, assent or disapproval, cooperativeness or resistance, through their bonding and affiliative functions (5–7). Routinized speech formulas form a large part of daily talk, communicating “beliefs, wants, wishes, preferences, norms, and values.” [(8), p. 239].

## Neurolinguistic background

The early impetus for recognizing the role of formulaic expressions (FEs) in speaking arose from observations in aphasia, using the term “automatic speech.” Starting with J. Hughlings Jackson in the nineteenth century (9, 10), clinicians with exposure to aphasia noted that fixed, holistic, known utterances are well-preserved despite severe language impairment [e.g., (11–17)]; these clinical observations were confirmed by systematic surveys (18–21). Early categories of “automatic” serial speech (counting and days of the week) have been greatly expanded to cover a very large domain. FEs are utilized to communicate in aphasic speech (22) and they play a key role in rehabilitation (23–25).

Examination of monologs from persons with left hemisphere (LH) damage and aphasia reveal high proportions of FEs, while right hemisphere (RH) damage is associated with significantly lower proportions (26, 27). Baldo et al. (28) also reported a trend toward fewer FEs in elicited responses in RH damaged speech than healthy speakers. Formal testing of persons with aphasia supported a preserved ability for FEs (29–31).

Persons with Alzheimer's disease (AD) speak with a preponderance of FEs throughout the progression of the disease; AD leaves the basal ganglia essentially intact for a considerable time (32). In contrast, Parkinsonian disease (PD) arises from impaired subcortical motor nuclei. Experimental studies confirmed that AD speakers' proportions of FEs are higher than healthy speakers, while PD speakers show deficient output (33–36).

## Functional imaging and the dual process model of language

The few functional imaging studies dealing with FEs have yielded contradictory results (This review focuses on production and does not include studies of novel metaphor). Using a precursor (^133^Xe) of PET, Larsen et al. (37) studied subjects at rest or while counting or reciting the weekdays. Rest values were subtracted from speaking values. For subjects who had the LH studied, there were significant task differences in two of four frontal regions. For subjects whose RH was studied, there were no differences in these regions. Interpretations were problematic because no direct left-right comparisons were possible, the normalization was different for left and right data sets, and task subtraction was employed.

Bookheimer et al. (38) used oxygen-labeled water with PET to study serial-months and the Pledge of Allegiance. Syllable repetition and an oral-motor task were included with a resting state. Using subtraction, the data from the non-propositional tasks were contrasted with data from the rest state. Of the 24 brain regions with blood flow increases, 14 were in the LH while 10 were in the RH. The results regarding functional lateralization were thus not definitive.

Using PET, counting and recitation of nursery rhymes were contrasted with spontaneous monologs (39). All tasks resulted in activation of left hemisphere frontal and temporal sites. This study relied on multiple, complex and simple additions and subtractions of images, lending complexity to interpretation.

Finally, in another PET study, healthy subjects produced animal names, vocalized syllables, and counting. Instead of subtraction, a partial least squares analysis was used (40). Three significant latent variables were identified: one for naming and syllables, with left anterior area predominating over right; a second for naming in bilateral anterior areas, and a third, associated with counting, involved RH and subcortical sites (41). Unlike the previous studies reviewed, these results corresponded to clinical observations, whereby even the most severely aphasic individuals can count.

We report a PET imaging study examining FEs, recently performed in our laboratory, using a complementary approach to activation methods: performance-based analysis. This method explores factors that contribute to cerebral lateralization for language (42, 43). The approach determines if there is a linear combination of brain regions that is predictive of performance during scanning. It is a fundamentally different approach to brain-behavior relationships as it does not rely on group mean differences or task contrasts. Rather, it identifies relationships between individual differences in performance and individual differences in brain activity. This method has consistently yielded functional profiles that are compatible with clinical observations (44).

**Figure 1:**
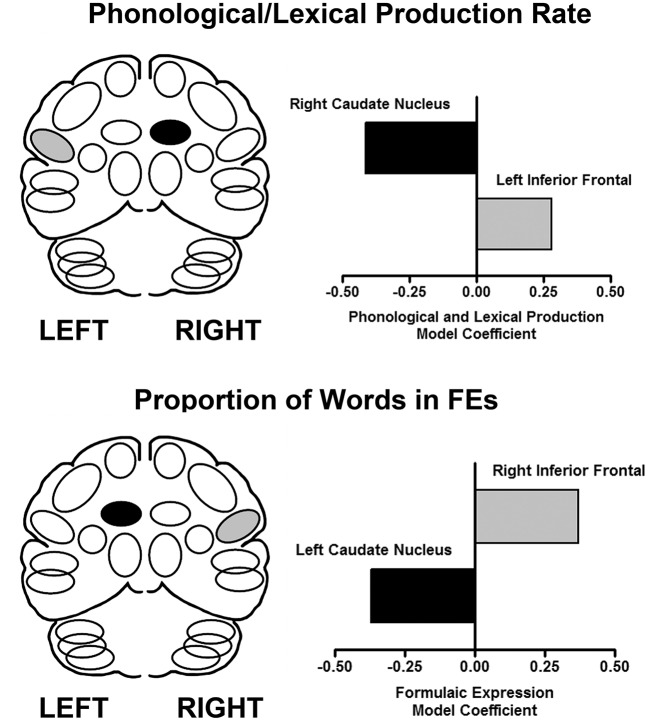
The results of performance-based analyses identifying relationships between brain regions that predict syllable and word production rate (top), and the proportion of words in FEs (bottom) using a multiple linear regression analysis. The X axis represents the multiple linear regression weights obtained in this analysis. On the left are schematic views of the predictor regions (light fill is an increase, dark fill is a decrease). On the right are graphical representations of the regression weights for the brain regions predictive of the respective expressive language measures in the linear regression model (47).

Speech samples (monologs, syllables and words) produced during scanning were recorded for acoustic and linguistic analyses. From monologs, FEs were quantified as the proportion of FE words in the total word count. Based on previous studies (45, 46), multiple regions were measured for the inferior frontal area and the caudate, bilaterally. The results are presented in Figure 1. Using a multiple linear regression analysis, the predictors of speech rate showed that as syllable and word production rates increased, blood flow increased in the left inferior frontal region and decreased in the right caudate. In contrast, the predictive model for the proportion of FEs in the monologs was a complementary pattern of cortical-subcortical interaction. As the proportion of FEs in the monologs increased, blood flow increased in the right inferior frontal region and decreased in the left caudate (47). This laterality profile is consistent with the effects of RH damage on the expression of FEs.

## Discussion

Formulaic expressions naturally carry an affective load. Idioms, proverbs, and other conventional expressions communicate a large range of positive and negative affects, implied within their non-literal meaning. In studies of persons with unilateral lesions and progressive neurological disease, it was observed that formulaic language relies on a cooperation between the cortical RH and subcortical nuclei. Performance based analysis of cerebral blood flow measured during formulaic and propositional speech identified predictive, complementary patterns corresponding with these two modes. Greater use of conversational speech formulas was associated with increased blood flow in the RH and reduced flow in the left caudate. Exemplars of propositional speech were significantly associated with the opposite pattern.

Known characteristics of the brain systems modulating formulaic as contrasted with grammatical language are compatible with the proposed dual model of language [e.g., (48–51)]. The RH specializes (52, 53) in empathy (including “theory of mind”) (54–59), affect and emotional experiencing (60–62), social-context based meanings and pragmatic competence (28, 63–68), diffuse lexical processes (69–71), personal familiarity (72, 73), and holistic configurations (74–76).

The basal ganglia stores and processes overlearned motor gestures. The characteristics of subcortical structures, shown to be important in FE production, include modulating routinized motor and verbal gestures (77, 78), including grammatical elements (79, 80) and recited speech (81, 82). Basal ganglia impairment interferes with normal production of FEs (36).

**Figure 2:**
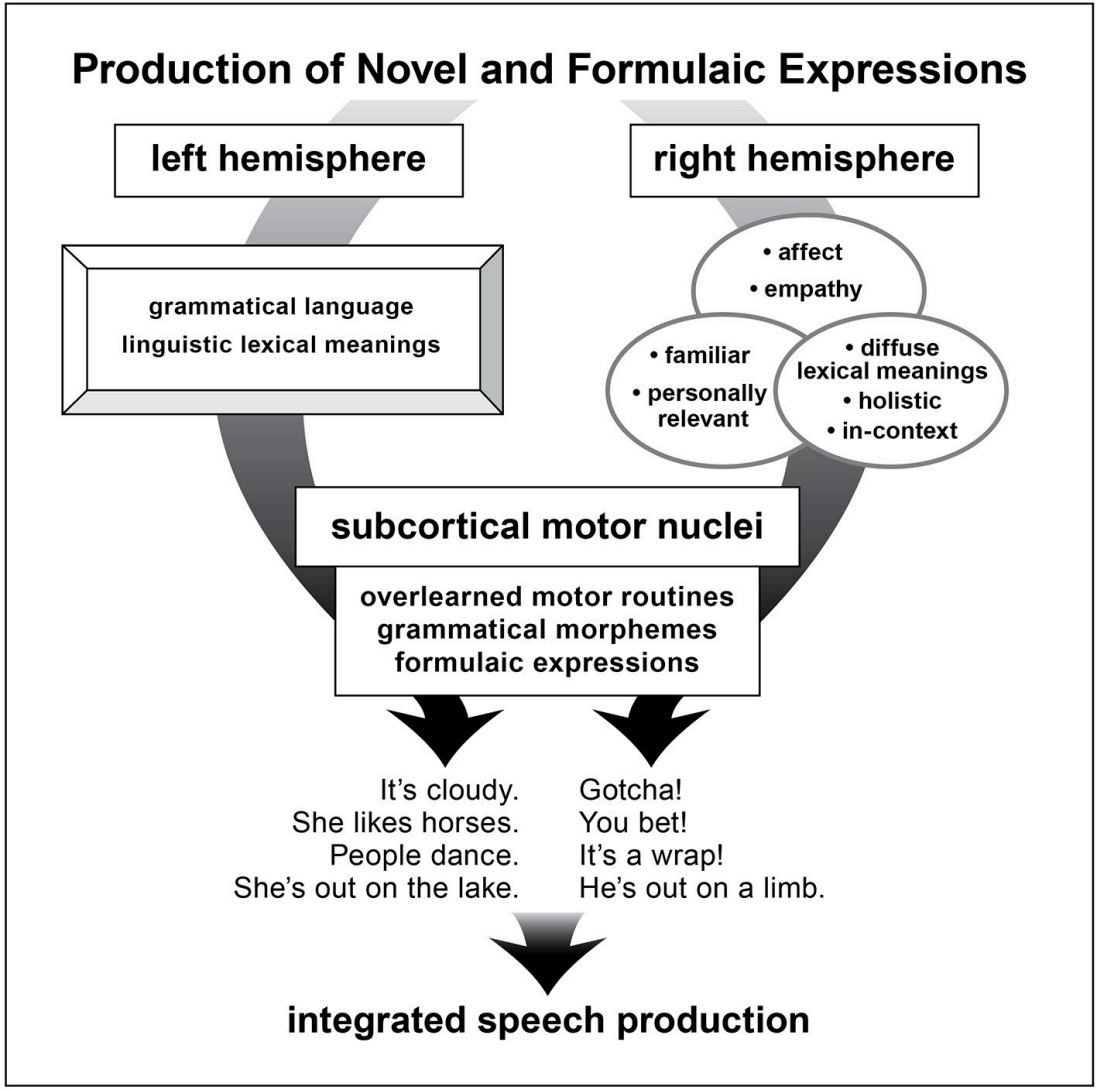
A schematic depiction of brain structures underlying production of novel and formulaic utterances as proposed in the dual process mocel of language.

Both of these structures, RH and basal ganglia, in their intrinsic functionality are well-suited to the properties of FEs (see Figure 2).

In the dual processing language model, two distinctive modes of language competence exist: formulaic and grammatical (83–87). These language modes have different intrinsic characteristics and rely on disparate cerebral systems. Further studies can look toward uncovering the cerebral switching mechanisms that allow for smooth integration of these two modes in fluent speech. Recognition of the dual process of language competence has important implications for our understanding of first language acquisition, second language learning, and clinical rehabilitation of language disorders.

## Author contributions

DS wrote major portions of paper, partly designed and contributed to experimental study reported in the paper. JS wrote portions of paper, partly designed and contributed to experimental study reported in paper, analyzed functional imaging results.

### Conflict of interest statement

The authors declare that the research was conducted in the absence of any commercial or financial relationships that could be construed as a potential conflict of interest. The reviewer CT and handling Editor declared their shared affiliation.
